# Uses of Cotton Seed

**Published:** 1889-05

**Authors:** 


### USES OF COTTON SEED.

Was there ever, says the *Banker's Monthly*, such a history as that of the cotton seed? For seventy years despised as a nuisance, and burned or dumped as garbage, then discovered to be the very food for which the soil was hungering, and reluctantly admitted to the rank of utilities, shortly afterwards found to be nutritious food for beasts as well as for soil, and thereupon treated with something like respect. Once admitted to the circle of farm industries, it was found to hold thirty-five gallons of pure oil to the ton, worth in its crude state $14 to the ton, or $40,000,000 for the whole crop of seed. But then a system was devised for refining the oil up to a value of one dollar a gallon, and the frugal Italian placed a cask of it at the root of every olive tree, and then defied the Borean breath of the Alps. And then experience showed that the ton of cotton seed was a better fertilizer and a better stock food when robbed of its thirty-five gallons of oil than before, that the hulls of the seeds made the best of fuel for feeding the oil-mill engine, that the ashes of the hulls scooped from the engine's draught had the highest commercial value as potash, and that the "refuse" of the whole made the best and purest soap stock, to carry to the toilet the perfumes of Lubin or Colgate.

